# Optimizing the Rheological and Textural Properties of Chapatti Enriched with House Crickets (*Acheta domesticus*) Flour Using Hydrocolloids by an I-Optimal Design

**DOI:** 10.3390/foods11213467

**Published:** 2022-11-01

**Authors:** Habiba Khatun, Siebe Lievens, Ruben Smets, Mohammad Akhtaruzzaman, Mik Van Der Borght, Johan Claes

**Affiliations:** 1 Research Group for Insect Production and Processing, Department of Microbial and Molecular Systems, Faculty of Engineering Technology, KU Leuven, Kleinhoefstraat 4, 2440 Geel, Belgium; 2 Department of Food Science and Nutrition, Faculty of Engineering, Hajee Mohammad Danesh Science and Technology University, Dinajpur 5200, Bangladesh; 3 Toxicological Centre, Department of Pharmaceutical Sciences, Faculty of Pharmaceutical, Biomedical and Veterinary Sciences, University of Antwerp, Universiteitsplein 1, 2610 Wilrijk, Belgium; 4 Institute of Nutrition and Food Science, University of Dhaka, Dhaka 1000, Bangladesh

**Keywords:** chapatti, *Acheta domesticus*, cricket flour, hydrocolloids, I-optimal design, dough rheology, textural properties

## Abstract

The fortification of food with edible insect flour can improve its nutrition profile, but also affect its techno-functional characteristics. In this study, an I-optimal design was applied to improve the rheological and textural properties of wheat flour chapatti containing 10% cricket (*Acheta domesticus*) flour. More specifically, the impact and optimal addition of hydrocolloids (carboxymethyl cellulose, hydroxypropyl methylcellulose, guar gum and xanthan gum) and water content were studied. For all the responses, the model and model terms were highly significant and showed the different impact of the hydrocolloids on the rheological properties. To evaluate the predictive power of the models, two sets of optimal process settings were chosen: one based on dough properties, and another on baked chapatti. For both sets, the actual responses were in the range of predicted responses for almost all properties. In addition, it was shown that using the settings based on dough properties, the actual responses were not significantly different from the control chapatti, whereas for the settings based on baked chapatti, there were differences in terms of the extensibility of both dough and chapatti. Thus, the I-optimal design is suitable to optimize the dough properties and the baked chapatti when enriching chapatti with cricket flour.

## 1. Introduction

Edible insects are gradually being explored as a potential alternative for traditional livestock and future food due to their high nutritional potential and environmental sustainability to cope with the increasing global protein demand [1,2,3,4,5]. Nevertheless, the acceptability of edible insects is not widespread globally due to cultural factors and disgust sensitivity towards insects. Invisible inclusion of edible insects, typically as a powder in a staple food product, could be one of the effective strategies to accelerate the acceptability [6]. Several researchers, however, showed that incorporation of edible insects in different foods, more specifically wheat flour based baked products, changed the techno-functional properties. Cappelli et al. (2020) reported for bread that a higher (15%) addition of house cricket (*Acheta domesticus*) or mealworm larvae (*Tenebrio molitor*) powder increased the dough tenacity and reduced dough extensibility which resulted in a decreased bread volume [7]. Osimani et al. (2018) investigated wheat flour bread with the addition of cricket flour at 10–30% and found that the technological properties of dough and bread were negatively correlated with the addition of cricket flour [8]. In another research, Azzollini and colleagues (2018) found that textural properties of wheat flour extruded snacks enriched with 10% mealworm (*T. molitor*) larvae flour were similar to that of control while the snacks enriched with 20% mealworm flour exhibited poor texture [9]. Moreover, Cabuk (2021) reported that softness and volume of wheat flour muffin were reduced by the inclusion of 15% grasshopper (*Locusta migratoria*) flour or mealworm (*T. molitor*) flour [10]. Thus, the effect of edible insect flour depends on the type of insect species, amount of insect flour added, and type, formulation, and ingredients of the food products. Thereby, the possibilities of incorporating a specific amount of edible insects in a food product without altering the functional properties cannot be generalized. The dilution of gluten content due to the substitution of wheat flour with edible insects’ flour might cause the alteration in the rheology and textural properties of the wheat flour-based dough and corresponding baked products. Since gluten is responsible for the viscoelastic properties of dough, the supplementation of wheat flour with non-gluten flour appears to interfere with the viscoelastic behavior of the dough, and thus eventually the final product quality [11].

To improve the gluten network properties different hydrocolloids have been widely used in wheat flour and gluten free baked products. For instance, hydroxypropyl methylcellulose (HPMC), xanthan gum and carboxymethyl cellulose (CMC) were reported to increase the specific volume and softness of potato flour based steamed bread [12]. Azeem et al. (2021) found that xanthan gum improved the bread quality in terms of higher specific volume and lower hardness, while working with sweet potato flour-wheat bread [13]. Further, sensory attributes and textural properties of bread were found to be improved with the addition of CMC (1–2%) or guar gum (2%) by Previtali et al. (2014) when wheat flour was supplemented with lentil flour (10–25%) as a protein source [14]. In addition, Ghodke et al. (2007) investigated the effects of adding guar gum, HPMC, CMC and kappa- carrageenan on whole wheat flour chapatti and found that the tensile force was reduced by the addition of guar gum, HPMC, and CMC up to 0.75% while tensile force increased at 1% addition of the specified hydrocolloids [15].

As such, extensive research has been performed on the effect hydrocolloids on wheat flour breads or gluten free bread. However, research on utilizing hydrocolloids in chapatti (an unleavened flatbread) dough and/or baked chapatti is still limited. Chapatti is a staple diet in the Asian subcontinent that act as a major source of nutrition. A research on increasing nutrition of chapatti by the addition of crickets flour showed that 5% addition was possible without altering the techno-functional properties, while an increased hardness and reduced extensibility of chapatti at higher content of cricket flour (10–15%) were observed [16]. Whereas inclusion of high amount of cricket flour can facilitate further nutritional improvement in chapatti. Therefore, this research aimed to improve the techno-functional properties of chapatti with a high amount of cricket flour (10%) using hydrocolloids. The application of hydrocolloids in a chapatti containing insect flour has not been investigated yet, while cricket flour, being rich in protein and fat, can interact differently with hydrocolloids and wheat starch and gluten. In addition, the impact of hydrocolloids cannot be generalized due to their diversified nature, structure, chain length, and molecular weight. The combination of hydrocolloids with new protein sources can further alter its functional and textural properties [17,18]. It should also be noted that more water is required to obtain a well-developed dough when adding hydrocolloids due to their higher water absorption capacity [11]. Thus, an I-optimal mixture design was employed to optimize a suitable combination of hydrocolloids (CMC, HPMC, guar gum, and xanthan gum) and water in the wheat flour dough containing 10% (flour weight) cricket (*A. domesticus*) flour. The optimization was performed to obtain comparable rheological and textural properties of wheat-cricket flour dough and baked chapatti to that of control sample (100% wheat flour). After performing the experimental runs according to the design of experiment (DoE), two confirmation tests were conducted to test the prediction power of the developed models.

## 2. Materials and Methods

### 2.1. Design of Experiment

For the design of this study five experimental factors, from X_1_ to X_5_ (representing the independent factors water, carboxymethylcellulose (CMC) hydroxypropyl methylcellulose (HPMC), guar gum, and xanthan gum, respectively) and two coded levels for each factor (low (−1) and high (+1)) were selected based on a preliminary screening test (results not shown), as can be seen in Table 1, while the amount of wheat flour (90%) and cricket flour (10% of the wheat flour weight) were kept constant. For the mixture design, the sum of all added hydrocolloids was maximum 2% of flour weight. The aim of this study was to find a suitable combination of water and hydrocolloids which will result in a dough and baked chapatti comparable to the control (wheat flour) chapatti. Hence, an I-optimal design was employed to find an optimum range of factors which will yield the responses within an acceptable range. Since 5 factors were used, 27 experimental runs were required (Table 2) to estimate all parameters and interactions. Additionally, constraints were put on the hydrocolloids amount. All combinations are allowed, but the total hydrocolloid amount can be maximum 2% of flour weight as the screening study (data not shown) revealed that inclusion of hydrocolloids above 2% did not exert positive effects. Finally, two confirmation tests were employed according to the optimal prediction settings of water and four hydrocolloids generated by the software JMP Pro 15. One optimal setting was based on dough properties and the other was based on the properties of baked chapatti.

The regression model was used to calculate the predicted response as shown in Equation (1), where Y is the predicted response, β_0_ is the intercept of the function, β_i_ is the linear coefficient of X_i_, β_ii_ is the quadratic coefficient X_i_, β_ij_ is the interaction coefficient of X_i_ and X_j_, and ϵ represents the error term.

(1) $$y\;{=\beta }_{0}{+\beta }_{1}X_{1}{+\beta }_{2}X_{2\;}{+\beta }_{3}X_{3\;}{+\beta }_{4}X_{4\;}{+\beta }_{5}X_{5\;}+\overset{5}{\underset{i=1}{\sum }}\beta_{\mathrm{ii}}X_{i}^{2}+\overset{5}{\underset{i=1}{\sum }}\overset{5}{\underset{j>i}{\sum }}\beta_{\mathrm{ij}}X_{i}X_{j}+\epsilon$$

### 2.2. Sample Collection and Preparation

Wheat flour (AllBak) was purchased from a wholesaler (Jan Gevers BVBA) in Mol, Belgium. House crickets (*Acheta domesticus*) were purchased from Nusect (Ledegem, Belgium). Cricket flour was prepared according to the procedure described by Khatun et al. (2021) [16]. Briefly, immediately after collecting, the crickets were kept at refrigeration temperature for 24 h after which the crickets were blanched and dried in a forced-air oven (UFB500, Memmert, Büchenbach, Germany) for 17 h at 65 °C. Afterwards, the crickets were ground using a kitchen grinder (DPA141, Moulinex, France) and sieved (mesh size < 1.18 mm) to obtain a fine powder. Four hydrocolloids, i.e., carboxymethylcellulose (CMC), hydroxypropyl methylcellulose (HPMC), guar gum, and xanthan gum were provided by Caldic Ingredients Benelux B.V., Belgium.

### 2.3. Rheological Properties of Dough

#### 2.3.1. Dough and Baked Chapatti Preparation

All the doughs were prepared according to the proportion of hydrocolloids and water as suggested by the DoE (Table 2), while wheat flour (90%), cricket flour (10%), and table salt (NaCl) (2.0% of flour weight) were constant for all the runs. To make the dough, first, all dry ingredients were mixed properly. Then, water was added and mixed to dough using a kneading machine (Kenwood Major Titanium KMM020, New Lane, England) at speed 3 for 5.0 min. The same dough was used for the analysis of biaxial extensibility (after resting for 5 min) and viscoelastic properties (after resting for 11 min) of dough and baked chapatti texture (after resting for 30 min).

Chapatti was prepared according to the method described by Khatun et al. (2021) [16]. A pasta sheeter (Model KAX 980 ME, Kenwood, Italy) was used to make the chapatti sheet of approximately 1.9 mm thickness which was then cut into a dimension of 12 cm x 10 cm. A tortilla maker (Princess, Tilburg, The Netherlands) was used to bake the chapatti at 215 ± 2 °C for 90 s on one side and for 120 s on the other side. The chapatti texture was analyzed after cooling for 7 min at room temperature (25 ± 2 °C).

#### 2.3.2. Dynamic Oscillatory Measurements

The dynamic rheology of the formulated dough was measured by a rheometer (model MCR 301, Anton Paar, Ostfildern, Germany) using parallel serrated plates (25 mm diameter) with a strain sweep from 0.01 to 100% at a frequency of 1 Hz as described by Khatun et al. (2021) [16]. When applying a strain sweep, the stress value required to obtain a specific strain was correlated with the storage modulus (G′, stored energy or elastic behavior) and the loss modulus (G″, dissipated energy or viscous behavior) in the dough. One measurement from each dough was obtained for all the experimental runs. For the confirmation tests, the mean of three measurements from three separate dough were obtained.

#### 2.3.3. Dough Extensibility

The biaxial extensibility of dough was measured using a texture analyzer (Stable Micro system TA.XTplus, Godalming, Surrey, England) with a tortilla/pastry rig (HPD/TPB) as described by Khatun et al. (2021) [16]. Briefly, after mixing, the dough was rested for 5 min and sheeted to a thickness of ≈1.9 mm by a pasta sheeter (Model KAX 980 ME, Kenwood, Treviso, Italy). Immediately after sheeting, the measurement was conducted by placing the dough on the heavy-duty platform. The mean of two measurements from one dough was obtained for all the experimental runs. For the confirmation tests, the mean of six measurements from three separate dough were obtained. The results were expressed as the maximum deformation force and the extensibility at maximum force. 

### 2.4. Chapatti Texture

Uniaxial extensibility of baked chapatti was measured by a texture analyzer (TA.XTplus, Stable Micro system, UK) using a tensile grip (A/TG) as described by Khatun et al. (2021) [16]. Briefly, after baking and cooling, the chapatti was cut at 7.0 cm × 3.5 cm dimension from the center and placed between two clamps of the texture analyzer at the distance of 22 mm using a load cell of 5 kg (speed of 1 mm/s). The force to extend the chapatti strip 1 mm and the distance of extension at maximum force were recorded. The force to extend the chapatti strip for 1 mm was considered as chapatti hardness. The mean of two measurements from one baked chapatti were obtained for all the experimental runs. For the confirmation tests, the mean of two chapatti per batch totaling six measurements from three batches were calculated. 

### 2.5. Statistical Analysis

JMP software (JMP Pro 15. SAS Institute Inc., Cary, NC, USA) was used to implement the I-optimal design and response surface analysis. Multiple regression analysis was conducted to model the variation of each response and to investigate the significant effects of all independent variables to the response. Analysis of variance (ANOVA) was performed at 5% level of significance to assess the model fitness. A backward elimination procedure was employed to select the significant (*p* ≤ 0.05) terms, which facilitates effect hierarchy. After removing non-significant terms, the models were fitted by accounting the coefficient of determination (*R*^2^) and adjusted coefficient of determination (Adj. *R*^2^) value. Afterwards, a prediction was used to choose optimal process setting for the desired response variable. Two sets of optimal process settings were chosen using the prediction profiler (JMP), one based on dough properties in terms of biaxial extensibility and viscoelastic properties, and another based on uniaxial extensional properties from the baked chapatti. Thereafter, a one-way ANOVA test was used to check the significant (*p* < 0.05) difference between the results of all parameters of control and experimental dough and chapatti during the confirmation tests.

## 3. Results and Discussion

### 3.1. Model Fitting

Twenty-seven experiments were conducted according to the design produced by the I-optimal design of mixture experiments to determine the effect of water and four hydrocolloids on dough rheology (biaxial extensibility and viscoelasticity) and textural properties of baked chapatti (uniaxial extensibility). For all the response variables, the significant (*p* < 0.05) main effects, interaction, and quadratic effect of water and four hydrocolloids level were retained. The resulting model and model terms were highly significant (*p* < 0.0001) while the coefficient of determination (*R*^2^) and adjusted coefficient of determination (Adj. *R*^2^) were higher than 0.80 (Table 2), which indicates that the models were highly adequate to predict the responses [19].

### 3.2. Dough and Baked Chapatti Quality as Affected by Water and Hydrocolloids

**Figure 1:**
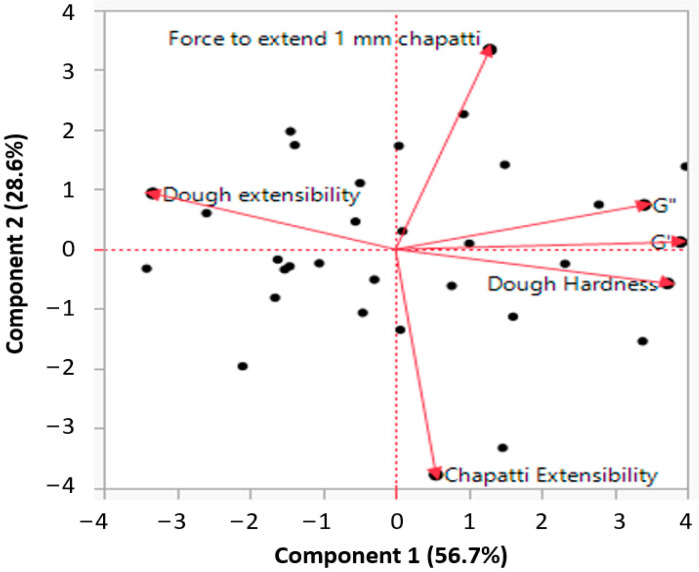
PCA biplot representing the rheological and textural properties of wheat-cricket flour dough and baked chapatti as affected by different hydrocolloids and water content.

A biplot from the principal component analysis (PCA) of dough and baked chapatti prepared from wheat-cricket flour properties by 27 runs is presented in Figure 1. The PCA result shows that a total 85.3% of the variations in obtained data have been explained by two principal components (PC1, 56.7%; and PC2, 28.6%). 

PC1 has been shown to be largely characterized by dough properties while PC2 was characterized by baked chapatti properties. Thus, PCA revealed the disparities among the wheat-cricket flour dough and baked chapatti properties as affected by different proportion of water and hydrocolloids type. Furthermore, a significant positive correlation was found between dough hardness and viscoelastic properties G′ and G″ (r = 0.88 and r = 0.71). As expected, dough hardness and dough extensibility showed a strong negative correlation (r = −0.78) which is clearly shown by PCA plot. Moreover, with the increase of chapatti hardness, its extensibility was reduced.

### 3.3. Biaxial Extensibility of Dough

#### 3.3.1. Dough Hardness

The most important characteristics of dough, force to extend the dough (hardness of dough) and extensibility of the dough at deformation have been extracted from the biaxial extensibility test. The prediction expression produced by the design is shown in Equation (2). From this expression, it can be seen that all the factors applied in the formulation of chapatti dough had a significant (*p* < 0.05) linear effect on the dough hardness.

(2) $$Y_{\mathrm{dough}\;\mathrm{hardness}}=2.53\;-\;0{.38\;X}_{1}+0{.23\;X}_{2}+0{.29\;X}_{3}+0{.38\;X}_{4}+0{.75\;X}_{5}-\;0{.18\;X}_{1}X_{2}$$

The most pronounced positive linear effect was shown by xanthan gum (X_5_), followed by guar gum (X_4_), HPMC (X_3_) and CMC (X_2_), while water had a negative linear effect on the dough hardness. Although CMC had a positive linear effect on dough hardness, it showed a significantly negative interactive effect when combined with water. The increase of dough hardness by xanthan gum could be attributed to its electrostatic interaction with gluten proteins. On the other hand, guar gum can increase dough hardness by interacting with starch and gluten through hydrogen bonds [20,21]. Furthermore, hydrocolloids can form hydrophilic complexes with gluten, thus increasing the dough strength [22]. In addition to these interactions between hydrocolloids and wheat flour, it can also be hypothesized that hydrocolloids interact with the cricket proteins, thereby increasing the dough hardness. However, further research is required to prove this hypothesis.

#### 3.3.2. Dough Extensibility

For dough extensibility, the model and model terms were highly significant (*p* < 0.0001) and the coefficient of determination (*R*^2^) and adjusted coefficient of determination (Adj. *R*^2^) value were 0.88 and 0.85 respectively (Table 2). The coefficient of estimation of dough extensibility (Equation (3)) showed that water (X_1_) exerted a significant (*p* < 0.05) positive linear effect while guar gum (X_4_) and xanthan gum (X_5_) had a significant (*p* < 0.05) negative linear effect on dough extensibility, with xanthan gum (X_5_) being more pronounced.

(3) $$Y_{\mathrm{dough}\;\mathrm{extensibility}}\;=\;55.96\;+\;8.31\;X_{1}\;-\;2.60\;X_{4}\;-\;15.59\;X_{5}\;+\;3.48\;X_{1}X_{4}\;+\;3.56\;X_{1}X_{5}$$

Significantly positive interaction effects on dough extensibility of water with guar and xanthan were also found. Farbo et al. (2020) found a reduced extensibility at 0.5% but increasing extensibility at 1.0% concentration of xanthan gum on wheat-cassava composite dough. However, such quadratic effect on dough extensibility by xanthan was absent in our study (Equation (3)) [23]. It can also be seen that CMC and HPMC did not exert any significant effect on dough extensibility, which is contrary to the findings reported by Smitha et al. (2008) who reported an increasing effect of HPMC on wheat flour dough extensibility [24]. A possible explanation for this contradictory finding might be the higher water holding capacity of cricket protein/flour compared to wheat flour that might create a stiffer conformation in the dough making it less extensible [25,26].

**Figure 2:**
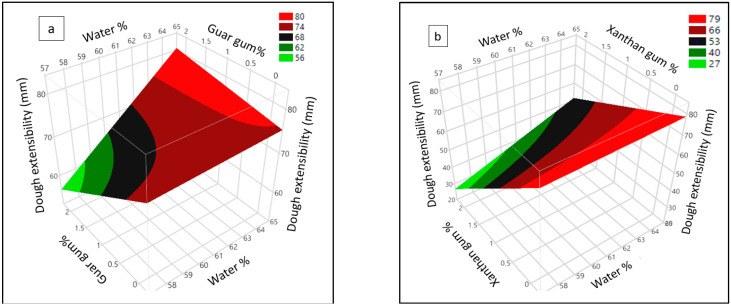
Surface plot showing interaction of water with (**a**) guar gum and (**b**) xanthan gum on dough extensibility containing wheat and cricket flour.

The surface plot (Figure 2a) clearly shows an interaction effect of water and guar gum, indicating at lower water content, with increasing guar gum, dough extensibility was reduced, while at higher water content dough, with increasing guar gum, extensibility was increased. However, at higher water content the lowering effect of xanthan gum on dough extensibility was less pronounced (Figure 2b) than lower water content. The presence of sufficient water in the dough system could mitigate the synergistic competition of hydrocolloids, starch, gluten, and insect protein for water, which eventually rendered the dough with greater extensibility without rupturing. This is supported by the fact that, in a wheat dough system, the available water can modify/develop a uniform and stable gluten–starch network [27].

### 3.4. Viscoelastic Properties of Dough

**Figure 3:**
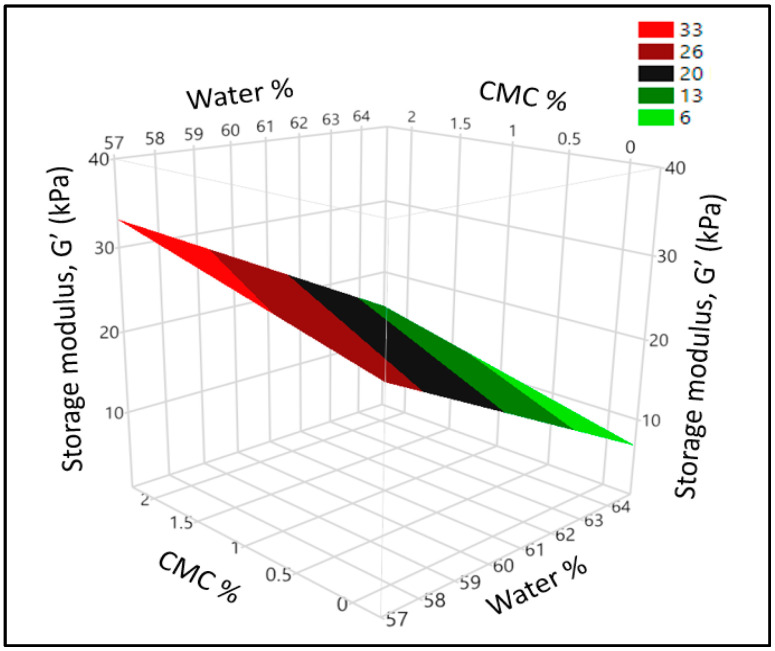
Surface plot showing the effect of water and CMC on the storage modulus (G′) of wheat-cricket flour dough.

Characterization of the fundamental rheology of dough requires studying the effect of small stresses on the viscoelastic properties which has been reported to relate with gas retention capacity as well as final bread quality. For chapatti making, gas retention is not crucial but moderate viscoelasticity is required for rolling the dough into a thin (1.5–2.0 mm) sheet [28]. In our study, the storage modulus (G′) was higher than the loss modulus (G″) for all the dough indicating predominant elastic behavior while there was no crossover in the studied range of the amplitude sweep. The statistical expression of regression coefficients showed that all the factors had significant (*p* < 0.05) main effects on storage modulus (G′) and loss modulus (G″) (Table 2) but no interaction or quadratic effect of the factors was observed (see Equations (4) and (5)). The addition of hydrocolloids increased the storage module (G’) while water has an opposite effect on the storage modulus (G′) as shown in Figure 3. The reduction of G′ with increasing water content is already established in wheat flour dough and composite dough systems due to the dilution effect of water on it [29].

(4) $$Y_{G\prime }=42.69\;-\;6.92{\;X}_{1\;}+3.36{\;X}_{2}+4.58{\;X}_{3}+7.38{\;X}_{4}+10.96{\;X}_{5}$$

(5) $$Y_{G\prime \prime }=15.82\;-\;2.32{\;X}_{1}+2.11{\;X}_{2}+2.16{\;X}_{3}+2.88{\;X}_{4}+2.55{\;X}_{5}$$

A more pronounced effect in both moduli was observed by xanthan gum (X_5_) and guar gum (X_4_) indicating stronger and less extensible dough with more elastic interaction which is in line with dough hardness and extensibility (Section 3.3.1 and Section 3.3.2). Xanthan gum can establish electrostatic interactions, more entanglements with gluten and crosslinking with starch that can contribute to dough strength. Moreover the side groups of xanthan gum can form single or double helix rods that further contribute to the rigidity of the resulting dough [30,31,32], while long, soluble, and rigid chains with higher hydrodynamic volume of guar gum might be responsible for its higher dynamic moduli [33]. As such, hydrocolloids and starch along with gluten and cricket protein possibly augmented the elasticity (G′) of the dough as viscoelastic characteristic of dough is strongly dependent on water and protein content [34]. Therefore, the interaction of gluten network and other dough components and their rheological behavior could be affected by several concurrent characteristics, including ionic character, the size of polysaccharide chains and conformation, as well as water availability in the dough system [35].

### 3.5. Uniaxial Extensibility of Chapatti 

Tensile deformation of chapatti has been found to successfully measure the texture of baked chapatti. The extensibility of chapatti, measured as the distance up to which chapatti is pulled apart without rupturing, is used to determine the freshness of chapatti [28]. 

#### 3.5.1. Chapatti Hardness

From the fitted model (Equation (6)), it was derived that all the factors had significant (*p* < 0.05) main effects on chapatti hardness, while quadratic effects of water (X_1_), guar (X_4_) and xanthan gum (X_5_) were also significant. Further, significant (*p* < 0.05) interaction effects of water with HPMC (X_3_), of guar gum with HPMC and xanthan were found on chapatti hardness.

(6) $$Y_{\mathrm{Force}\;\mathrm{to}\;\mathrm{extend}\;\mathrm{chapatti}}=8.87\;-\;0{.61X}_{1}+0.87\;X_{2}+1.52\;X_{3}+4.93\;X_{4}+4.03\;X_{5}+0{.71\;X}_{1}^{2}+0.69\;X_{1}X_{3}$$

**Figure 4:**
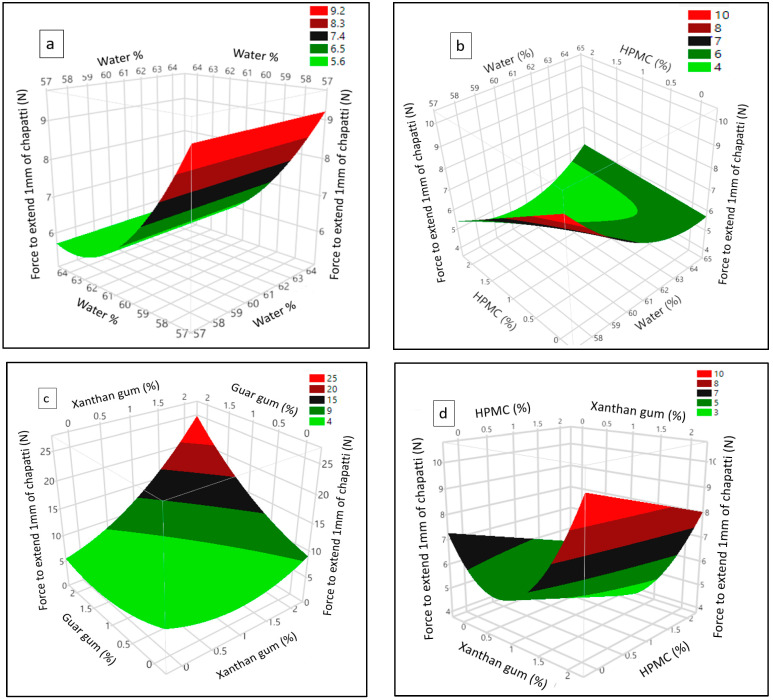
Response surface plot showing (**a**) quadratic effect of water; the interactions of (**b**) water and HPMC, (**c**) xanthan and guar gum, and (**d**) xanthan gum and HPMC on wheat-cricket flour chapatti hardness expressed as force to extend 1 mm.

The quadratic effect of water indicated that chapatti hardness was reduced with the increase of water until 63% but further increase of water increased the chapatti hardness as visualized in Figure 4a. Further, the interaction of water and HPMC (Figure 4b) shows that at lower level of HPMC, with increasing water level chapatti hardness was reduced. However, at higher level of HPMC, increasing the water content first reduced chapatti hardness while a further increase of the water level produced a harder chapatti. 

Figure 4c shows the interaction effect of guar and xanthan gum. At a lower content of guar gum, the effect of xanthan on chapatti hardness was only limited, but at a higher level of guar gum, a small increase of xanthan drastically increased chapatti hardness and vice versa. A similar but less pronounced observation was made for the interaction of xanthan and HPMC (Figure 4d). 

HPMC, xanthan gum and CMC were reported to increase the specific volume and softness of wheat flour composite bread due to its higher dough viscosity that could confer a more stable starch-gluten network during baking [12,36]. Xanthan gum was also reported to be responsible for higher entanglement with α-helix conformation of gluten. This entanglement might be intensified by cricket protein in which α-helix is the most prominent secondary protein conformation [32,37]. 

It was interesting to notice the interaction effect of guar-xanthan and HPMC-xanthan on the baked chapatti (Equations (6) and (7)) while such interaction between biopolymers was absent in the dough system (Equations (2) and (3)). It can be hypothesized that these interactions were developed due to the heat treatment during the baking stage where intermolecular interactions could occur between the heat-disordered segment of xanthan gum and the backbone of guar gum. This is supported by Khouryieh et al. (2006), who observed a strong interaction effect of guar and xanthan gum at 80 °C in an aqueous system, while cooling down to 25 °C left xanthan gum in a more disordered conformation [38]. Further, thermal-induced aggregation of cricket protein with hydrocolloids as well as gluten protein might have intrigued the interaction of biopolymers in baked chapatti [37]. 

#### 3.5.2. Chapatti Extensibility

The coefficient of estimation for chapatti extensibility shows that the model terms are highly significant (*p* < 0.0001) while *R^2^* and adjusted *R^2^* were 0.816 and 0.735 respectively indicating good fitting of the model (Table 2). From the prediction expression (Equation (7)), it can be seen that all the factors except CMC were found to have a significant (*p* < 0.05) positive main effect on chapatti extensibility. While water (X_1_) showed a negative quadratic effect, a positive quadratic effect was exerted by HPMC (X_3_).

(7) $$Y_{\mathrm{Chapatti}\;\mathrm{extensibility}}=4.03\;+\;0.23\;X_{1}\;+\;1.95\;X_{3}\;+\;0.81\;X_{4}\;+\;2.37\;X_{5}\;-\;0.33\;X_{1}^{2}\;+\;0.65\;X_{3}^{2}\;+\;1.56\;X_{3}X_{5}\;+\;0.60\;X_{4}X_{5}$$

**Figure 5:**
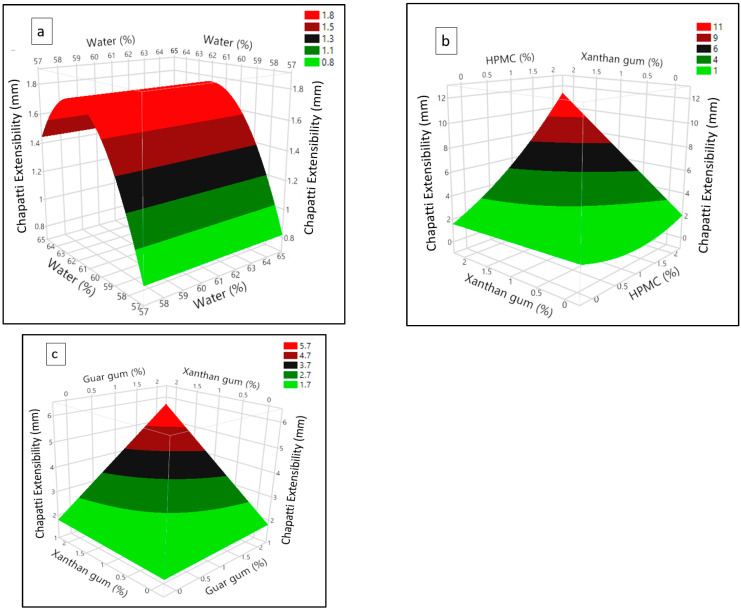
Surface plot depicting (**a**) quadratic effect of water, and interaction effect of (**b**) xanthan-HPMC, (**c**) xanthan-guar on wheat-cricket flour chapatti extensibility.

In addition, significant positive interactions of xanthan (X_5_) with HPMC (X_3_) and guar gum (X_4_) were also observed on chapatti extensibility. The quadratic effect of water (Figure 5a) shows that increasing the water content first increased the chapatti extensibility which reduced thereafter. This can be explained as excessive water dilutes the gluten network rendering the baked chapatti less extensible. Both the HPMC and xanthan showed a higher increasing effect on chapatti extensibility, with xanthan being more pronounced, as can be deduced from its higher coefficient (Equation (7)). The uncoiling of the xanthan chain during heating could have allowed ionic bonding with protein resulting in an increase of elasticity of baked wheat-cricket flour chapatti [31].

It is clearly depicted by the surface plot (Figure 5b), that at higher level of HPMC, small increase of the xanthan gum content can result in a pronounced increase of the chapatti extensibility as shown by the much steeper curve. It is possible that the higher water binding ability and thickening properties of HPMC and xanthan provided greater stability to the dough during baking [36]. This might have contributed to a prominent increase in the chapatti extensibility without rupturing. Similarly, greater chapatti extensibility can be obtained by a small increase of guar gum at a higher level of xanthan (Figure 5c). 

### 3.6. Confirmation Tests

Two confirmatory tests were conducted, as shown in Table 3, one test was based on the optimal factor settings for dough properties (dough hardness, dough extensibility and elastic modulus G′) while the parameter settings for other test were based on baked chapatti texture (chapatti hardness and extensibility). In the first test, xanthan gum was excluded as from the model of dough harness (Equation (2)) and dough extensibility (Equation (3)) it was observed that the linear effect of xanthan can increase the hardness and decrease the extensibility of dough while a dough with medium hardness and higher extensibility is, generally, suitable to produce a desirable chapatti [39]. The results from the confirmation test showed that all the responses except dough hardness were within the prediction limit range indicating the good fitness of the models (Table 3). The dough hardness was slightly higher (1.28 ± 0.05 N) than that of predicted range (0.98–1.24 N), but it was not different from control sample 2, which took into account the ageing of the wheat flour. The results also revealed that the ternary mixture of CMC/HPMC/guar resulted in a lowering effect on elastic or storage modulus (G′). This is in line with the reducing interactive effect of CMC and water on dough hardness as shown by the prediction expression (Equation (6)). CMC might have outweighed the increasing effect of guar and HPMC on G’ by the ternary effect with HPMC and guar, as CMC is less elastic and more viscous in nature than other gums [40]. This is further supported by Jo and Yoo (2018), who concluded that a small amount of CMC could have a substantial reducing effect in elastic properties in the ternary mixture of CMC/xanthan/locust bean gum [29]. The obtained dough extensibility was found to be comparable with the control sample (wheat flour dough). This can be explained by the absence of a significant increasing or decreasing effect of CMC and HPMC on dough extensibility while guar gum might have increased dough extensibility by an interaction effect with water (Equation (3)).

The ternary mixture of CMC/HPMC/guar resulted in a comparable chapatti hardness with the control chapatti. This result is in agreement with Ghodke Shalini and Laxmi (2007), who reported that guar gum had a pronounced reducing effect on chapatti hardness followed by CMC and HPMC up to a content of 0.75% [15]. The chapatti extensibility was within the predicted interval although it was lower than that of the control chapatti, but higher than control sample 2. Ghodke Shalini and Laxmi (2007) reported that guar gum had most increasing effect on wheat flour chapatti extensibility followed by HPMC and CMC at 0.75%, 0.75% and 0.50%, respectively [15]. However, the effects of guar gum and HPMC are consistent, but no significant increasing effect of CMC was observed in our study, as mentioned earlier. This difference might be attributed to the addition of cricket flour in our study that rendered a different dough composition. 

Another confirmatory test based on the texture of baked chapatti was performed by excluding CMC and including xanthan gum (Table 3). The results showed that, like the previous confirmation test, all the responses were almost within the range of the predicted values for optimal settings of all the factors showing a good performance of the DoE approach. For the dough extensibility and chapatti hardness, the results were slightly outside the predicted range showing either slightly higher or lower values. These small deviations of the results from the predicted values might be due to the storage effect of wheat flour as this experiment was conducted after one month of the first test, which could be confirmed by the results with control sample 2 (Table 3). Storage of wheat flour can increase the water absorption capacity of wheat flour and thus affect the dough rheology and baked chapatti texture. However, this type of deviations from optimal predictions are not completely uncommon while working with DoE [41,42]. 

Including xanthan gum in the mixture design of HPMC/guar/xanthan gum resulted in higher dough strength (hardness and G′) than the ternary mixture of CMC/HPMC/guar gum (Table 3). Strengthening of wheat flour dough due to addition of xanthan gum and guar gum was also reported by other researchers [35,36,43]. However, in our study the interaction of gluten and cricket protein with xanthan through ionic bonding as well as sulfide bonding can contribute to dough strength [44]. Dough extensibility produced by the mixture of HPMC/guar gum/xanthan gum was slightly higher than that of predicted value but comparable to the control dough (Table 3), indicating the ternary mixture of HPMC/guar gum/xanthan gum with suitable water content can provide a desirable effect on dough extensibility. The chapatti hardness produced by HPMC/guar/xanthan mixture was also comparable to that of the control chapatti (Table 3). Measured chapatti extensibility was within the predicted interval and comparable with the control value, which confirmed the positive effect of HPMC/guar/xanthan mixture on chapatti extensibility (Table 3). As such, it is apparent that not only the concentration, structure, and hydrophobicity of the hydrocolloids, but also the dough components and their interactions with hydrocolloids have a direct or indirect influence on the rheology and textural attributes of chapatti. 

## 4. Conclusions

An I-optimal mixture design was employed to optimize the rheological and textural properties of wheat flour chapatti supplemented with 10% cricket flour (*Acheta domesticus*) using four hydrocolloids, namely carboxymethylcellulose (CMC), hydroxypropyl methylcellulose (HPMC), guar gum, and xanthan gum, and a variable water content. The obtained models showed that all the different hydrocolloids and water affected differently on the dough and chapatti rheology. Results showed that all the hydrocolloids had a positive influence on dough strength (dough hardness and G’). For dough extensibility, guar and xanthan gum showed a negative effect when used singly, while their interaction with water had a positive effect on dough extensibility, indicating a crucial function of water. In contrast, CMC and HPMC did not show any effect on dough extensibility. The quadratic effect of guar gum and xanthan gum could exert a softening effect on baked chapatti at low level while CMC and HPMC showed a hardening effect on baked chapatti with an increasing concentration. 

To test the predicting capacity of the models, two sets of optimal process setting were chosen using the obtained models. One was based on dough properties (ternary mixture CMC/HPMC/guar gum), and the other on the baked chapatti properties (ternary mixture HPMC/guar gum/xanthan gum). In both cases, water content was predicted by the models. Results revealed that for the settings based on dough properties, the properties of the obtained dough and of the baked chapatti were within the predicted range, and not significantly different from the control (100% wheat flour). The measured responses of the second confirmation test, with settings based on baked chapatti, were also well in agreement with the predicted responses, but with some deviations, especially in terms of dough extensibility and chapatti extensibility. Therefore, it can be concluded that the design of experiments approach proved to be a strong tool to optimize the dough properties and the baked chapatti when nutritionally enriching chapatti with cricket flour.

## Figures and Tables

**Table 1:** Experimental and coded (between brackets) factors settings for the I-optimal design used.

Factors (% of Flour Weight)	Range of Variables	
	Low (−1)	High (+1)
Water (X_1_)	58 (−1)	64 (+1)
Carboxymethylcellulose (CMC) (X_2_)	0 (−1)	2 (+1)
Hydroxypropyl methylcellulose (HPMC) (X_3_)	0 (−1)	2 (+1)
Guar gum (X_4_)	0 (−1)	2 (+1)
Xanthan gum (X_5_)	0 (−1)	2 (+1)

**Table 2:** Rheological and textural characteristics of dough and baked chapatti containing 10% cricket flour as influenced by water, CMC, HPMC, guar gum and xanthan gum.

Run	Water (%)	CMC (%)	HPMC (%)	Guar Gum (%)	Xanthan Gum (%)	Dough Hardness (N)	Dough Extensibility (mm)	Storage Modulus G′ (kPa)	Force to Extend 1 mm of Chapatti (N)	Chapatti Extensibility (mm)
1	58.04	0.34	0.00	0.00	1.66	2.49	45.92	41.14	9.57	1.50
2	63.91	0.00	0.00	0.00	0.00	0.79	74.95	11.98	5.26	1.70
3	61.01	0.00	1.00	0.00	0.00	1.16	73.94	20.75	5.64	1.92
4	61.02	0.00	0.00	0.00	1.00	1.55	50.00	34.86	4.97	2.03
5	61.04	0.41	0.40	0.40	0.39	1.74	69.39	27.17	4.39	2.16
6	60.96	1.00	0.00	0.00	0.00	1.11	74.99	20.07	6.28	2.02
7	64.00	0.00	1.72	0.28	0.00	1.32	74.97	20.05	5.36	1.78
8	61.02	1.00	0.00	1.00	0.00	1.51	74.96	27.31	4.77	1.85
9	58.03	0.00	0.74	0.00	0.73	2.00	54.68	37.63	6.03	1.75
10	61.03	0.40	0.40	0.40	0.39	1.55	67.16	27.26	6.20	1.57
11	58.01	0.72	0.00	0.70	0.00	1.16	66.86	28.48	7.32	1.04
12	61.04	0.00	0.00	1.00	0.97	2.38	52.51	40.42	6.16	2.64
13	64.01	2.00	0.00	0.00	0.00	1.20	74.99	18.91	8.18	1.26
14	61.13	0.00	0.00	1.00	0.00	1.11	74.89	21.67	4.52	1.94
15	64.00	0.00	0.00	0.00	2.00	1.70	54.15	29.21	7.64	1.91
16	57.94	0.00	0.00	2.00	0.00	1.99	61.00	39.90	7.56	1.72
17	64.02	1.00	0.00	0.00	1.00	1.61	62.43	22.63	5.05	1.99
18	61.00	0.40	0.40	0.40	0.39	1.55	73.35	24.75	6.44	1.57
19	64.01	0.00	0.20	1.80	0.00	1.36	74.90	23.07	5.60	1.85
20	57.96	0.00	0.00	0.00	0.00	1.25	74.99	20.02	8.64	1.18
21	58.04	0.00	1.00	1.00	0.00	1.65	64.24	34.73	7.15	1.16
22	61.01	1.00	1.00	0.00	0.00	1.37	74.93	25.50	6.37	1.23
23	57.98	1.79	0.01	0.00	0.19	1.72	72.58	30.08	8.86	1.17
24	63.99	0.70	0.70	0.00	0.00	0.94	74.99	14.48	6.53	1.49
25	58.01	0.00	2.00	0.00	0.00	1.57	69.79	32.49	5.14	2.03
26	60.98	0.00	1.00	0.00	1.00	2.13	56.67	29.13	4.57	3.36
27	64.02	0.00	0.00	0.75	0.71	1.05	68.95	17.09	4.45	2.55
Model										
F-Value (Model)						19.39 *	32.047 *	30.49 *	10.32 *	10.03 *
F-Value (Lack of fit)						NA	0.1539 ^ns^	NA	NA	0.9404 ^ns^
*R* ^2^						0.8533	0.8841	0.8789	0.8832	0.8168
Adjusted *R*^2^						0.8093	0.8565	0.8501	0.7977	0.7354

* *p* < 0.0001, ^ns^ = not significant at *p* < 0.05, NA= not applicable, because the model is saturated, meaning that there are as many estimated parameters as there are observations.

**Table 3:** Actual responses of dough and baked chapatti prepared with wheat flour (control) and wheat- cricket flour obtained by predicted optimal setting of all factors.

Responses	Dough Hardness (N) *	Dough Hardness (N) **	Dough Extensibility (mm) *	Dough Extensibility (mm) **	Storage Modulus (G’) (kPa) *	Storage Modulus (G’) (kPa) **	Chapatti Hardness (N) *	Chapatti Hardness (N) **	Chapatti Extensibility (mm) *	Chapatti Extensibility (mm) **
Predicted *** responses	[0.98; 1.24]	[1.43; 1.54]	[72.39; 77.02]	[65.07; 68.25]	[16.02; 20.35]	[22.96; 26.32]	[4.94; 6.04]	[4.02; 5.12]	[1.21; 1.72]	[1.97; 2.31]
Actual responses	1.28 ± 0.05 ^b^	1.49 ± 0.11 ^c^	74.97 ± 0.02 ^a^	73.0 ± 2.16 ^a^	17.50 ± 0.64 ^a^	21.69 ± 1.26 ^b^	6.05 ± 0.53 ^a^	6.39 ± 0.16 ^a^	1.55 ± 0.06 ^a^	2.21 ± 0.36 ^b^
Control 1 †	0.98 ± 0.15 ^a^		74.98 ± 0.01 ^a^		16.50 ± 0.64 ^a^		7.49 ± 1.60 ^a^		2.02 ± 0.22 ^b^	
Control 2 ‡	1.25 ± 0.19 ^b^		74.90 ± 0.74 ^a^		14.26 ± 1.70 ^a^		7.07 ± 1.68 ^a^		1.09 ± 0.47 ^a^	

Actual results are presented as mean ± standard deviation of three replicates. Different letters in a column mean statistically different values (*p* < 0.05), * Factors settings (X_1_ = 62.50, X_2_ = 0.25, X_3_ = 0.80, X_4_ = 0.10, X_5_ = 0.00, % of flour weight) for predicted responses and actual responses based on dough properties of chapatti. ** Factors settings (X_1_ = 62.00, X_2_ = 0.00, X_3_ = 0.30, X_4_ = 0.50, X_5_ = 0.50, % of flour weight) for predicted responses and actual responses based on baked chapatti texture. *** The values between the brackets indicate the range of each response from minimum to maximum limit. † Control 1 refers to the dough and chapatti prepared at the time of conducting 27 runs and used in the DoE that represents the reference sample throughout the text. ‡ Control 2 represents the dough and baked chapatti prepared at the time of confirmation test performed after 1 month of storage of wheat flour.

## Data Availability

Data is contained within the article.
